# Changes in the Neural and Non-neural Related Properties of the Spastic Wrist Flexors After Treatment With Botulinum Toxin A in Post-stroke Subjects: An Optimization Study

**DOI:** 10.3389/fbioe.2018.00073

**Published:** 2018-06-15

**Authors:** Ruoli Wang, Johan Gäverth, Pawel A. Herman

**Affiliations:** ^1^Department of Women's and Children's Health, Karolinska Institutet, Stockholm, Sweden; ^2^Department of Mechanics, Royal Institute of Technology, Stockholm, Sweden; ^3^KTH Biomex Center, Royal Institute of Technology, Stockholm, Sweden; ^4^Functional Area Occupational Therapy & Physiotherapy, Karolinska University Hospital, Stockholm, Sweden; ^5^Department of Computational Science and Technology, Royal Institute of Technology, Stockholm, Sweden

**Keywords:** spasticity, botulinum toxin A, stretch reflex, muscle mechanical properties, neuroflexor, motoneuron pool

## Abstract

Quantifying neural and non-neural contributions to the joint resistance in spasticity is essential for a better evaluation of different intervention strategies such as botulinum toxin A (BoTN-A). However, direct measurement of muscle mechanical properties and spasticity-related parameters in humans is extremely challenging. The aim of this study was to use a previously developed musculoskeletal model and optimization scheme to evaluate the changes of neural and non-neural related properties of the spastic wrist flexors during passive wrist extension after BoTN-A injection. Data of joint angle and resistant torque were collected from 21 chronic stroke patients before, and 4 and 12 weeks post BoTN-A injection using NeuroFlexor, which is a motorized force measurement device to passively stretch wrist flexors. The model was optimized by tuning the passive and stretch-related parameters to fit the measured torque in each participant. It was found that stroke survivors exhibited decreased neural components at 4 weeks post BoNT-A injection, which returned to baseline levels after 12 weeks. The decreased neural component was mainly due to the increased motoneuron pool threshold, which is interpreted as a net excitatory and inhibitory inputs to the motoneuron pool. Though the linear stiffness and viscosity properties of wrist flexors were similar before and after treatment, increased exponential stiffness was observed over time which may indicate a decreased range of motion of the wrist joint. Using a combination of modeling and experimental measurement, valuable insights into the treatment responses, i.e., transmission of motoneurons, are provided by investigating potential parameter changes along the stretch reflex pathway in persons with chronic stroke.

## Introduction

Spasticity is a motor symptom commonly seen after a lesion of the upper motor neuron, e.g., after stroke, spinal cord injury, or cerebral palsy. Clinically, spasticity is defined as a velocity-dependent increase in tonic stretch reflex with exaggerated tendon jerks as a component of upper motor neuron lesions (Lance, 1980). In current practice, it is commonly measured subjectively by rotating a joint and estimating the resistance according to an ordinal scale, such as the Modified Ashworth Score (MAS) (Ashworth, 1964). MAS is usually employed to evaluate the clinical effectiveness of Botulinum toxin type A (BoNT-A), one of the most popular anti-spastic treatments aimed at reducing focal spasticity. The effects of BoNT-A include the decreased resistance to muscle stretch and increased the passive range of movement (Ozcakir and Sivrioglu, 2007). However, it is generally agreed that MAS has limited reliability and reproducibility (Fleuren et al., 2010), and is not able to discriminate between the underlying neural (stretch reflex) and non-neural contributions of resistant joint torque (Bakheit et al., 2001). Thus, alternative methods have been developed to objectively quantify resistant joint torque and its components (Harlaar et al., 2000; Mirbagheri et al., 2001; van der Helm et al., 2002). Several laboratories have developed custom-built apparatuses to examine joint stiffness in terms of underlying neural and mechanical properties, mostly at the ankle joint (Mirbagheri et al., 2001; Lorentzen et al., 2010; de Vlugt et al., 2013; Blanchette et al., 2015) and elbow joint (Ju et al., 2000; Calota et al., 2008). Recently, Lindberg et al. (2011) have developed a mechanical device that functions as an instrumented version of the MAS test to quantify spasticity of wrist and finger flexors and the test is convenient for daily clinical practice. The instrument (NeuroFlexor, Aggero MedTech AB, Solna Sweden) passively extends the wrist at two different velocities. A force transducer measures the resistance throughout the movements. The real-time analysis method, i.e., the NeuroFlexor Method (NF-Method), can separate the contributions to the measured resistance into a passive elastic component, passive viscous component, and a neural component. The validity of the NF-Method was demonstrated in chronic stroke patients through the strong correlation between the neural component and the electromyography (EMG) activity of flexor carpi radialis (FCR), both across all subjects and in each subject during the nerve block test (Lindberg et al., 2011). Recent investigations have also demonstrated that the NF-Method is reliable (Gäverth et al., 2013) and sufficiently sensitive to detect changes in spasticity by intramuscular injections of BoNT-A in wrist and finger muscles (Gäverth et al., 2014). However, the NF-Method does not allow for identifying effects on detailed mechanical properties of the joint nor the transformation of the motoneuron pool and muscle spindles.

Several theories have been proposed to explain the cause of spasticity, including decreased threshold or increased gain of the stretch reflex, hyper-excitability of the α-motor neuron and hypersensitivity of muscle spindles (Powers et al., 1988; Katz and Rymer, 1989; Sheean, 2002). The most common techniques of assessing neurophysiological outcomes after BoNT-A treatment include surface electromyography (EMG) (Hesse et al., 1996; Pandyan et al., 2002; Albani et al., 2010), electrical nerve stimulation (ENS) (Pauri et al., 2000; Sheykh et al., 2005; Manca et al., 2010) and stretch-reflex threshold (SRT) (Stampacchia et al., 2004; Chen et al., 2005; Lee et al., 2008). In some studies, EMG activity is recorded during the passive or active movement, and the magnitude or timing of the EMG activity is used to quantify the effect of the BoTN-A (Burgen et al., 1949). However, EMG magnitude can be highly variable between days, and the lack of standardized normalization can potentially lead to a misleading interpretation of treatment efficacy (Phadke et al., 2012). In the ENS, electrical stimulation of a peripheral nerve is used to evoke the Hoffmann's reflex (H-reflex), M-wave, or F-wave. The responses of the stimulations have been used as a potential outcome of the treatment (Sheykh et al., 2005). Although the ENS is widely used, these electrically evoked responses often do not correlate with the responses evoked with mechanical activation of the reflex, i.e., muscle stretching (Leis et al., 1996; Morita et al., 1998). The SRT is usually defined as the smallest angle or the lowest velocity that elicits muscle activity during passive stretching, which offers another alternative to measure the neurophysiological effect of BoNT-A. The SRT can provide meaningful information on the sensitivity of the neurophysiological outcomes after BoNT-A as long as a standard movement velocity and intensity can be consistently delivered (Phadke et al., 2012). Regardless of the varied limitations of the abovementioned techniques, direct measurement of motoneuron, and biophysical properties of human is not possible (Hidler and Rymer, 1999). In order to investigate the potential pathophysiological mechanisms of spasticity, a forward neuromusculoskeletal model that accounts for dynamics of muscle spindles, motoneuron pools, muscle activation and musculotendon of the wrist flexors has therefore been developed recently (Wang et al., 2017). The model can describe the overall resistant behavior of the wrist joint during passive extension test of spasticity. By tuning the passive and stretch-related parameters to fit the measured torque, neural and non-neural related properties of the spastic wrist flexors were estimated (Wang et al., 2017). Furthermore, the model explicitly accounts for motoneuron pool and muscle spindle behavior unlike the aforementioned NF-Method. The validity of the proposed optimization scheme was demonstrated through good fit to data, overall robustness to fluctuations in parameters and agreement of available EMG recording and the model estimation in the chronic stroke survivors and healthy controls (Wang et al., 2017). In line with a previous study by Koo et al. (Koo and Mak, 2006), two motoneuron pool parameters (threshold and gain) in the model have been shown to be physiologically meaningful and capable of characterizing the overall transmission of the motoneuron pool of spastic muscles. The aim of this work is to demonstrate the feasibility of the proposed modeling and optimization framework for investigating the potential neurophysiological effects of BoTN-A treatment. It is established that intramuscular administration of BoTN-A results in a decreased output from neuromuscular junction by inhibiting the release of acetylcholine from presynaptic motor neurons (Jankovic, 2004). However, how the BoTN-A injection influences the transmission of the overall motoneuron pool has not yet been investigated, even though it is believed that the injection causes decreased neural excitations that can paralyze the spastic muscles (Jankovic, 2004).

All in all, the primary objective of this study is to evaluate the effect of the BoNT-A treatment on the potential spasticity-related manifestations in persons with stroke using the proposed modeling framework. The data for fitting the model were previously collected using NeuroFlexor apparatus (Gäverth et al., 2014). Although they were already used to investigate the sensitivity of the NF-Method to the changes in spasticity after the BoTN-A injection (Gäverth et al., 2014), the approach adopted in the current study allows for separating motoneuron pool and muscle spindle effects as they are explicitly accounted for in the model, and thus provides additional insights in the mechanisms of BoTN-A efficiency. The key finding was that stroke survivors exhibited decreased neural components at 4 weeks post BoNT-A injection, which returned to baseline levels after 12 weeks. Based on the model, the decreased neural component was predicted to stem from the increased motoneuron pool threshold.

## Method

### Subjects

A sample of 21 persons with chronic stroke (ST) (18 males/3 females, mean ± SD, age: 50 ± 12 yrs, body weight: 76 ± 13 kg) was selected from previous studies. All participants were recruited from a local rehabilitation clinic (Danderyd University Hospital, Department of Rehabilitation Medicine, Stockholm, Sweden) and received treatment of BoNT-A (Botox®, Allergan, Irvine, CA, USA) in one or more of their wrist and/or finger flexors after assessing by physicians (Table 1). Inclusion criteria were the following: (1) stroke > 6 months prior to inclusion, (2) spasticity in wrist and finger flexors, with a MAS ≥ 1, (2) at least 50° passive range of motion (ROM) at the wrist joint (20°Flexion to 30° extension), and (3) ability to understand and comply with instruction. The physician selected the muscles and the dose based on clinical experience and goal-setting. No additional treatment or hand training was prescribed. The participants were examined before and after intramuscular injections of BoNT-A in the spastic wrist and finger flexor muscles. The NeuroFlexor data was collected on 3 occasions: at baseline (maximum 2 weeks before treatment), 4 weeks after treatment, and 12 weeks after treatment. Four of the participants dropped out from the assessment at 12 weeks, due to illness, problems with transportation, or time constraints. The passive ROM of the wrist was also measured using a goniometer at each occasion. All participants gave written informed consent according to the Declaration of Helsinki. The study was approved by the Regional Ethics Committee, Karolinska Institutet, Stockholm, Sweden. The neuromusculoskeletal model and optimization framework was implemented using Matlab 2014b (The MathWorks, Inc., Natick, US).

**Table 1:** Clinical description of participants (FDS, flexor digitorum; FCR, flexor carpi radialis; FDP, flexor digitorum profundus; FCU, flexor carpi ulnaris).

**Subject**	**Age range (years)**	**Weight (Kg)**	**Lesion type**	**Post stroke time (Months)**	**BoNT-A units**	**Injected muscles**	**Modified ashworth score baseline**
S1	56–60	96	Haemorrhagic	123	90	FDS = 40, FCR = 50	1
S2	61–65	60	Ischemic	114	60	FDP = 30, FDS = 30	3
S3	51–55	75	Haemorrhagic	111	155	FDP = 35, FDS = 40 FCR = 40, FCU = 40	3
S4	41–45	77	Ischemic	114	60	FDP = 40, FDS = 20	4
S5	46–50	86	Ischemic	37	160	FDP = 40, FDS = 40 FCR = 40, FCU = 40	4
S6	56–60	82	Ischemic	55	50	FCR = 25, FCU = 25	4
S7	41–45	80	Haemorrhagic	10	100	FDP = 50, FDS = 50	0
S8	41–45	61	Haemorrhagic	28	85	FDP = 40, FDS = 45	3
S9	41–45	75	Ischemic	36	93	FDP = 25, FDS = 23 FCR = 20, FCU = 25	3
S10	46–50	82	Haemorrhagic	10	190	FDP = 40, FDS = 50 FCR = 50, FCU = 50	2
S11	51–55	39	Haemorrhagic	50	100	FDP = 50, FDS = 50	2
S12	61–65	71	Ischemic	12	100	FDP = 50, FDS = 50	3
S13	66–70	80	Ischemic	44	85	FDP = 35, FDS = 50	1
S14	21–25	75	Haemorrhagic	16	200	FDP = 50, FDS = 50 FCR = 50, FCU = 50	4
S15	56–60	69	Ischemic	7	140	FDP = 30, FDS = 30 FCR = 40, FCU = 40	3
S16	66–70	87	Ischemic	20	20	FDS = 20, FCR = 40	4
S17	46–50	75	Haemorrhagic	51	60	FDP = 40, FDS = 20	4
S18	61–65	78	Haemorrhagic	47	200	FDP = 50, FDS = 50 FCR = 50, FCU = 50	2
S19	46–50	84	Haemorrhagic	30	160	FDP = 40, FDS = 40 FCR = 40, FCU = 40	1
S20	46–50	100	Ischemic	43	100	FCR = 50, FCU = 50	2
S21	46–50	67	Haemorrhagic	23	190	FDP = 50, FDS = 50 FCR = 40, FCU = 50	2

### Experimental setup and protocol

**Figure 1:**
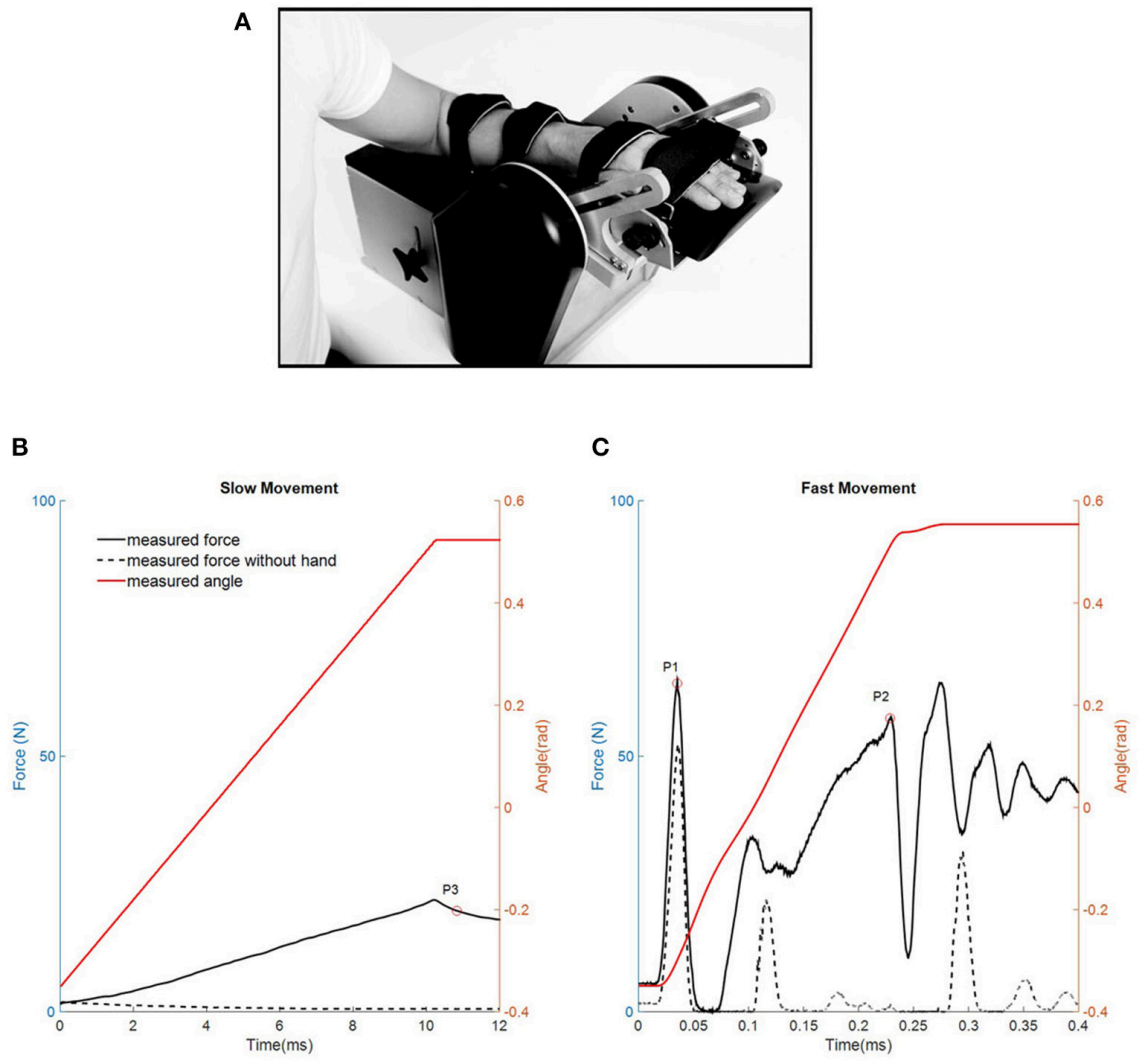
**(A)** The NeuroFlexor instrument (Photo printed with permission from Aggero MedTech AB, Stockholm Sweden). **(B,C)** Example measurements of resistant force profiles during slow **(B)** and fast movements **(C)** in one representative stroke patient. Red trace represents the angle of wrist movement (from flexion to extension). Black trace represents one resisting force trial and black dashed trace represents one resistant force trial when device runs without hand. Three time points are identified by the NeuroFlexor Method (NF-Method): P3 1 s after slow passive stretch; P1 the first peak and P2 the peak toward the end of the fast movement.

The resistance torque was measured using the NeuroFlexor that produced constant movements at a slow (5°s^−1^) and a fast (236°s^−1^) velocity (see details in Lindberg et al., 2011). The participants were seated comfortably with elbow in 90° of flexion and the hand securely fastened into the device with the wrist axis of rotation aligned with device (Figure 1A). In order to eliminate measurement variance, a clam and relax environment was carefully maintained during testing. The hand rested on the platform with the metacarpophalangeal joints in slight flexion and the fingers fully extended. Both the hand and arm were fastened using non-elastic velcro straps in order to ensure that movement could only occur at the wrist joint. The instrument performed slow and fast movements in a 50° ROM (20°Flexion to 30° extension) with a starting angle of 20° of palmar flexion. For each participant and test occasion, 5 slow movements and 10 fast movements were recorded. Resistance profiles were also obtained when the device ran empty (without hand; see an example data in Figures 1B,C) to enable the biomechanical model to isolate forces originating from the hand. As input to the neuromusculoskeletal model and optimization framework, the measured data of raw torque and angle were low pass filtered with a cut-off frequency of 10 Hz (second-order Butterworth filter).

### The NF-method

The NF-Method has been previously presented (Lindberg et al., 2011; Pennati et al., 2016), and it can separate the active muscle force component induced by the stretch reflex from the passive mechanical components. The method is briefly described below.

Three time points at the measured resistant force-time curve were defined specifically to estimate the inertial (IC), viscosity (VC), elasticity (EC), and neural components (NC): one point at the initial peak (P1) and one point at the later peak (P2) during the fast movement, and one point at fully stretched position (P3) during the slow movement.

Inertia component (IC) was defined as the force resisting the acceleration of the hand as:

(1) $$\begin{array}{r} \text{IC}=\text{ma} \end{array}$$

with *m* the mass of the hand and platform and *a* the acceleration.

Elasticity component (EC): The length –dependent elasticity was recorded at P3, 1s after the end of the slow movement, which included both the linear elasticity and nonlinear end range stiffness.

Viscosity component (VC): The early viscosity component was defined as the resisting force that remained after the inertia component had been subtracted from the initial peak of the total resistant force at P1.

(2) $$\begin{array}{r} \text{V}{\text{C}}_{\text{p}1}=\text{Total forc}{\text{e}}_{\text{p}1}-\text{IC} \end{array}$$

The late viscosity was estimated as:

(3) $$\begin{array}{r} \text{VC}=\left(\text{Total forc}{\text{e}}_{\text{p}1}-\text{IC}\right)\times 0.2 \end{array}$$

The neural component (NC) was estimated at P2 by subtracting the elasticity and viscosity component from the total force as:

(4) $$\begin{array}{r} \text{NC}=\text{Total forc}{\text{e}}_{\text{p}2}-\left(\text{EC}+\text{VC}\right) \end{array}$$

### Neuromusculoskeletal model

The full description of the neuromusculoskeletal model can be found in our previous study (Wang et al., 2015, 2017). The model is only briefly described here.

#### Musculotendon modeling

The musculotendon model was modified based on an lumped Hill - model described previously by Winters (Winters and Stark, 1985), which consisted of one lumped muscle actuator that represents synergistic wrist flexors and one passive unit. Wrist extensors were not included in the model due to their negligible activation during the passive wrist extension movement.

#### Equation of motion

The modeled wrist-resistant torque was determined according to dynamic equilibrium during the passive wrist extension movement:

(5) $$T_{m}=(J_{h}+J_{hp})\ddot{\theta }+T_{MA}+T_{PP}+T_{Gh}+T_{Ghp}$$

with *T*_*m*_ the modeled resistant moment, *J*_*h*_ and *J*_*hp*_ the moments of inertia of the hand segment and hand-plate, $\ddot{\theta }$ the angular acceleration, *T*_*MA*_ the torque generated by the lumped muscle actuator, *T*_*PP*_ the torque generated by the passive unit and *T*_*Gh*_ and *T*_*Ghp*_ the torques due to the gravity of the hand segment and hand-plate, respectively.

#### Passive unit

The passive unit in the model was considered as the passive viscoelastic elements of the muscle and connective tissues of the joint (Winters and Stark, 1985). Thereby, the elasticity component (EC) was an angle-dependent torque component of *T*_*PP*_, and the viscosity component (VC) was an angular velocity-dependent torque component.

(6) $$\begin{array}{r} EC=K_{p}\left(\theta -\theta_{0}\right)+k_{1}e^{k_{2}\left(\theta -\theta_{0}\right)-1} \end{array}$$

and

(7) $$\begin{array}{r} VC=B_{p}\overset{\cdot }{\theta } \end{array}$$

with *K*_*p*_ denoting the stiffness coefficient, *B*_*p*_ the viscosity coefficient, *k*_1_ and *k*_2_ the non-linear exponential coefficients, and θ_0_ the resting joint angle at the muscle-tendon slack length, such that *T*_*PP*_ is equal to zero.

#### Lumped muscle actuator

The lumped muscle actuator was modeled as a contractile element in series with a series elastic element. The joint moment-angle and moment-angular velocity relationships were implemented in the actuator model (Winters and Stark, 1985).

(8) $$\begin{array}{r} T_{MA}=af_{v}\left(\overset{\cdot }{\theta }\right)f_{l}\left(\theta \right) \end{array}$$

with *a* denoting the muscle activation, *f*_*v*_ the moment-angular velocity relationship, and *f*_*l*_ the moment-angle relationship. In passive wrist extension, muscle activation *a* was considered entirely induced by the stretch reflex of the wrist flexors. Therefore, the neural component (NC) was equivalent to *T*_*MA*_.

#### Muscle spindle modeling

The modified hybrid v^0.6^ model (Prochazka and Gorassini, 1998) was implemented to describe the firing characteristics of the muscle spindle in lengthening as a function of joint angle and angular velocity:

(9) $$\begin{array}{r} r\left(t\right)=G_{v}{\left(\overset{\cdot }{\theta }\left(t-\tau_{SRD}\right)\right)}^{0.6}+G_{l}\left(\theta \left(t-\tau_{SRD}\right)-\theta_{0}\right)+r_{0} \end{array}$$

with *r* denoting the Ia afferent (the primary afferent) firing rate, θ_0_ the rest joint angle, τ_*SRD*_ the stretch reflex delay, *G*_*v*_ and *G*_*l*_ the dynamic and static gains of the spindle model respectively, and *r*_0_ the background discharge rate of the Ia afferent neuron. τ_*SRD*_ and *r*_0_ were set to 30 ms and 6.45 imps^−1^ (Wilson et al., 1999).

#### Motoneuron pool modeling

The input-output relationship for the α-motoneuron pool was represented by a Gaussian cumulative distribution function (Fuglevand et al., 1993). The motoneuron pool transformation, i.e., the motoneuron neural excitation as a function of the muscle spindle firing rate, was modeled as a sigmoid function which ranges from 0 to 1,

(10) $$\begin{array}{r} u\left(t\right)=\frac{1}{1+e^{\frac{\mu -r\left(t\right)}{\sigma }}} \end{array}$$

with *u* denoting the neural excitation of the motoneuron pool, μ the muscle spindle firing rate at 50% motoneuron recruitment, and σ the standard deviation of cumulative distribution function.

The threshold and gain of the motoneuron pool of wrist flexors were further calculated based on the definition from a previous study (Koo and Mak, 2006). A threshold μ_0_ corresponds to the spindle-firing rate that results in neural excitation of 0.005, and gain *G*_0_ is defined in the following manner:

(11) $$\begin{array}{r} G_{0}=\frac{0.6}{r_{0.8}-r_{0.2}} \end{array}$$

with *r*_0.8_ and *r*_0.2_ denoting the muscle spindle firing rate at 80 and 20% of full motoneuron recruitment.

#### Muscle activation dynamics

Muscle activation dynamics was described as the process of converting neural excitation *u*(*t*) to muscle activation *a*(*t*) as a first order differential equation (Thelen, 2003).

### Optimization

A previous study indicated that the EMG activity of FCR was rarely observed in chronic stroke patients during the slow movement (Lindberg et al., 2011). Therefore, it was assumed that the resistant torque was entirely caused by the passive unit. Nonlinear least-squares (NLS) optimization was used to approximate the passive parameters ([*K*_*p*_, *B*_*p*_, *k*_1_, *k*_2_]) based on the experimental measurements of the resistant torque in the slow movement. A genetic algorithm (GA) (Golberg, 1989) was used to identify the optimal stretch reflex-related parameters ([*G*_*v*_, *G*_*l*_, μ, σ]) by minimizing the root-mean-square error (RMSE) between the measured and modeled resistant torque in part of the fast movement. Constraints were imposed on *G*_*v*_ and *G*_*l*_ to vary ±10% from the reported values (4.3 and 2.0 respectively).

### Data analysis and statistics

A set of trials (one slow movement, one fast movement, one slow movement without the hand and one fast movement without the hand) from each participant's NeuroFlexor measurements were randomly selected to calculate the EC, VC, and NC at each assessment occasion using both NF-Method and optimization, respectively. The same trials were also used for computing passive and stretch reflex related parameters using the neuromusculoskeletal model and optimization. The validity of the model was determined for each trial by the Variance Accounted For (VAF) (de Vlugt et al., 2010):

(12) $$\begin{array}{r} VAF=\left(1-\frac{\sum \varepsilon^{2}\left(t\right)}{\sum {T_{meas}\left(t\right)}^{2}}\right)\cdot 100\% \end{array}$$

with ε(*t*) denoting the error, i.e., the difference between the measured resistant torque, *T*_*meas*_(*t*) and the modeled resistant torque, *T*_*m*_(*t*), over *t* = 1, …, N samples. An extensive validity and reliability analysis of the used method and the estimated model parameters was performed previously (Gäverth et al., 2013; Wang et al., 2017).

Statistical analyses were performed using IBM SPSS Statistics (IBM Corp., Armonk, NY, USA). Descriptive statistics were expressed as median and range (or mean and standard deviation as appropriate). Spearman's rank order correlation test was used to investigate the correlation between components identified by the NF-Method and optimization, respectively. Differences over three time points in passive and stretch reflexed-related parameters were calculated using a linear mixed model with one subject effect: time, consisting of three time points (compound symmetry). A *post-hoc* pairwise comparison with Bonferroni adjustment was applied if the omnibus test was significant. Differences were considered statistically significant if *p*-value ≤ 0.05.

## Results

### Passive parameters

**Figure 2:**
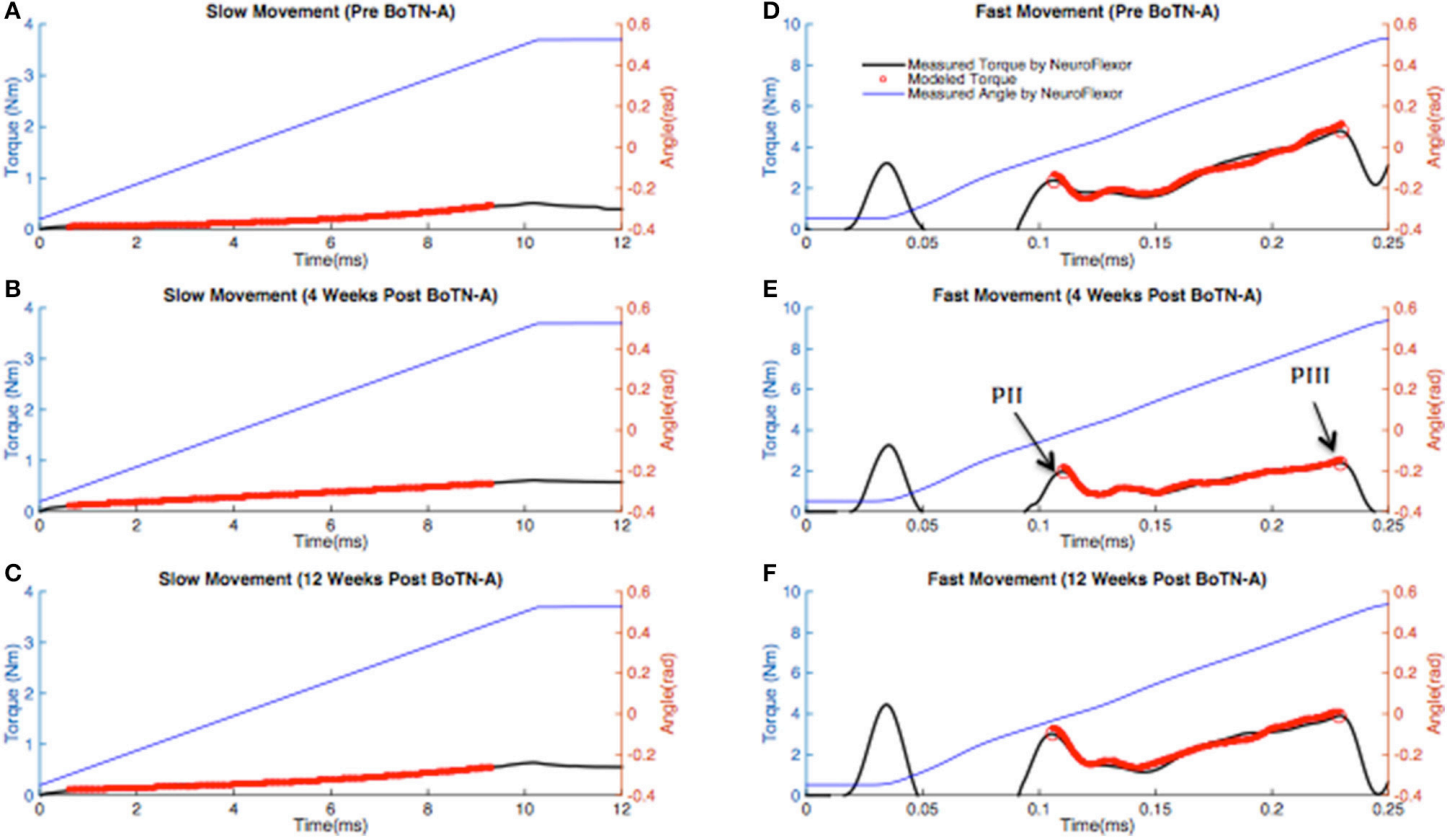
Measurements obtained with the NeuroFlexor and model based estimates of resistant torque for one representative stroke patient at three test occasions. The blue solid line represents the measured joint angle. The black solid line represents the measured resistant torque. The line with red circles represents the modeled resistant torque for the same subject. **(A–C)** In the slow movement, the wrist joint was passively extended from 20° (0.35 rad) to 30° (0.52 rad) of extension at 5°/s using a NeuroFlexor. A nonlinear-least squares optimization was used to predict passive parameters by fitting to the torque measured in the slow movement. **(D–F)** In the fast movement, the wrist joint was passively extended from 20° (0.35 rad) of flexion to 30° (0.52 rad) of extension at 236°/s (4.08 rad/s) using a NeuroFlexor. A genetic algorithm (GA) was used to estimate stretch reflex-related parameters. The optimization was only used to match data recorded in the period between PII to PIII.

As an example, Figures 2A,C shows the modeled and measured total resistant torque of one participant during the slow movement. It illustrates a good fit of the model. Consistent findings were observed in all participants. The VAF values of three assessment occasions were similar for optimization (pre BoNT-A: 95.2% ± 2.9%, 4 weeks post BoNT-A: 95.1% ± 2.9%, 12 weeks post BoNT-A: 93.1% ± 3.5%), which validated the generalization properties of the model fitted to individual participants. Compared to the baseline, there was a significant increase of non-linear coefficient *k*_1_ (*p* = 0.02) at 12 weeks post BoNT-A. In addition, a significant decrease of passive ROM (*p* = *0.01*) was determined at 12 weeks post BoNT-A. No significant changes in other passive parameters were found between test occasions (Table 2).

**Table 2:** Individual components, the passive and reflex related parameters (median and range) determined using modeling and optimization at baseline, 4 weeks and 12 weeks post BoNT-A injection.

**Median (Min, Max)**		**pre BoNT-A**	**4 weeks post BoNT-A**	**12 weeks post BoNT-A**
**Passive Range of Motion**	**(degree)**	65 (30, 85)	60 (10, 85)	55* **(20, 70)**
**Individual Component**	**NC (N)**	14.71 (5.54, 20.29)	7.90* **(2.00, 24.14)**	12.58 (0.16, 27.07)
	**EC (N)**	8.87 (1.85, 30.53)	9.22 (2.06, 25.92)	9.37 (2.24, 26.44)
	**VC (N)**	4.41 (0.43, 15.51)	4.33 (0.42, 15.28)	6.47 (0.43, 12.96)
**Passive Parameters**	***K**_*p*_* **(Nm** · **rad**^−1^**)**	0.80 (0.01, 3.24)	0.65 (0.05, 2.99)	0.69 (0.06, 4.01)
	***B**_*p*_* **(Nms** · **rad**^−1^**)**	0.13 (0.01, 0.35)	0.10 (0.01, 0.35)	0.15 (0.01, 0.30)
	***k**_1_* **(rad**^−1^**)**	0.07 (−2.59, 0.86)	0.13 (−0.84, 0.86)	0.28** **(**−**0.57, 4.51)**
	***k**_2_* **(rad**^−1^**)**	0.82 (−0.004, 4.33)	0.63 (−0.06, 6.45)	0.92 (−5.53, 4.17)
**Reflex Related Parameters**	***G**_*l*_*	2.09 (1.90, 2.10)	2.09 (1.90, 2.30)	2.09 (1.90, 2.10)
	***G**_*v*_*	4.24 (4.08, 4.50)	4.30 (4.10, 4.51)	4.40 (4.10, 4.50)
	μ	194.07 (150.00, 310.00)	280.83* **(150.30, 400.00)**	168.90 (150.00, 391.20)
	σ	16.09 (10.00, 79.8)	10.95 (10.00, 78.00)	18.90 (10.00, 77.80)
	*μ_0_*	97.05 (15.36, 285.14)	118.27* **(10.58, 246.53)**	60.83 (4.24, 235.62)
	***G**_0_*	0.013 (0.003, 0.022)	0.019 (0.003, 0.022)	0.011 (0.003, 0.022)

*Significant differences between baseline and post-treatment were illustrated in bold*.

* *= 0.01*.

** *p = 0.02*.

### Stretch reflex-related parameters

Due to measurement error during the period 0.05–0.09s of the recording and the latency of the reflex, the optimization was only performed for a selected part of the fast movement (from the second peak, PII, to the third peak, PIII, of the measured total resistant torque, Figure 2E), during which the angular velocity remained stable. As an example, Figures 2D,F shows the modeled and measured resistant torque of one participant during the fast movement. It also illustrates a good fit of the model to the data. Consistent findings were observed in all participants. The VAF values of the three assessment occasions were similar for optimization (pre BoNT-A: 90.1% ± 2.1%, 4 weeks post BoNT-A: 89.8% ± 2.2%, 12 weeks post BoNT-A: 89.6 ± 2.5%).

**Figure 3:**
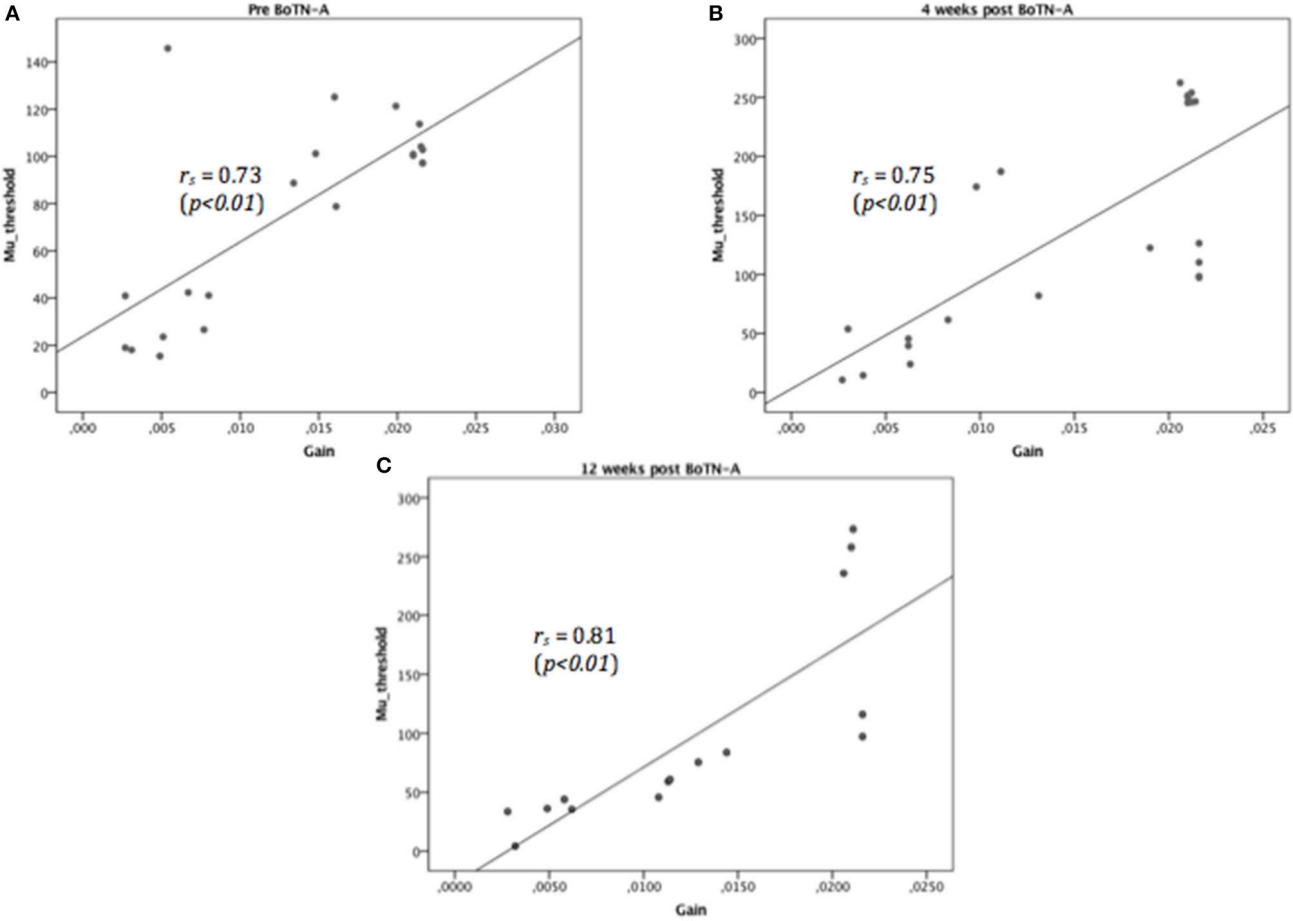
Highly correlated motoneuron pool parameters, threshold, μ_0_, and gain, *G*_0_, were determined at three test occasions: before BoTN-A treatment **(A)**, 4 **(B)**, and 12 **(C)** weeks after BoNT-A treatment. The solid lines represent the linear regression fit to the relationship of threshold and gain (*r*_*s*_ denotes the correlation coefficient).

**Figure 4:**
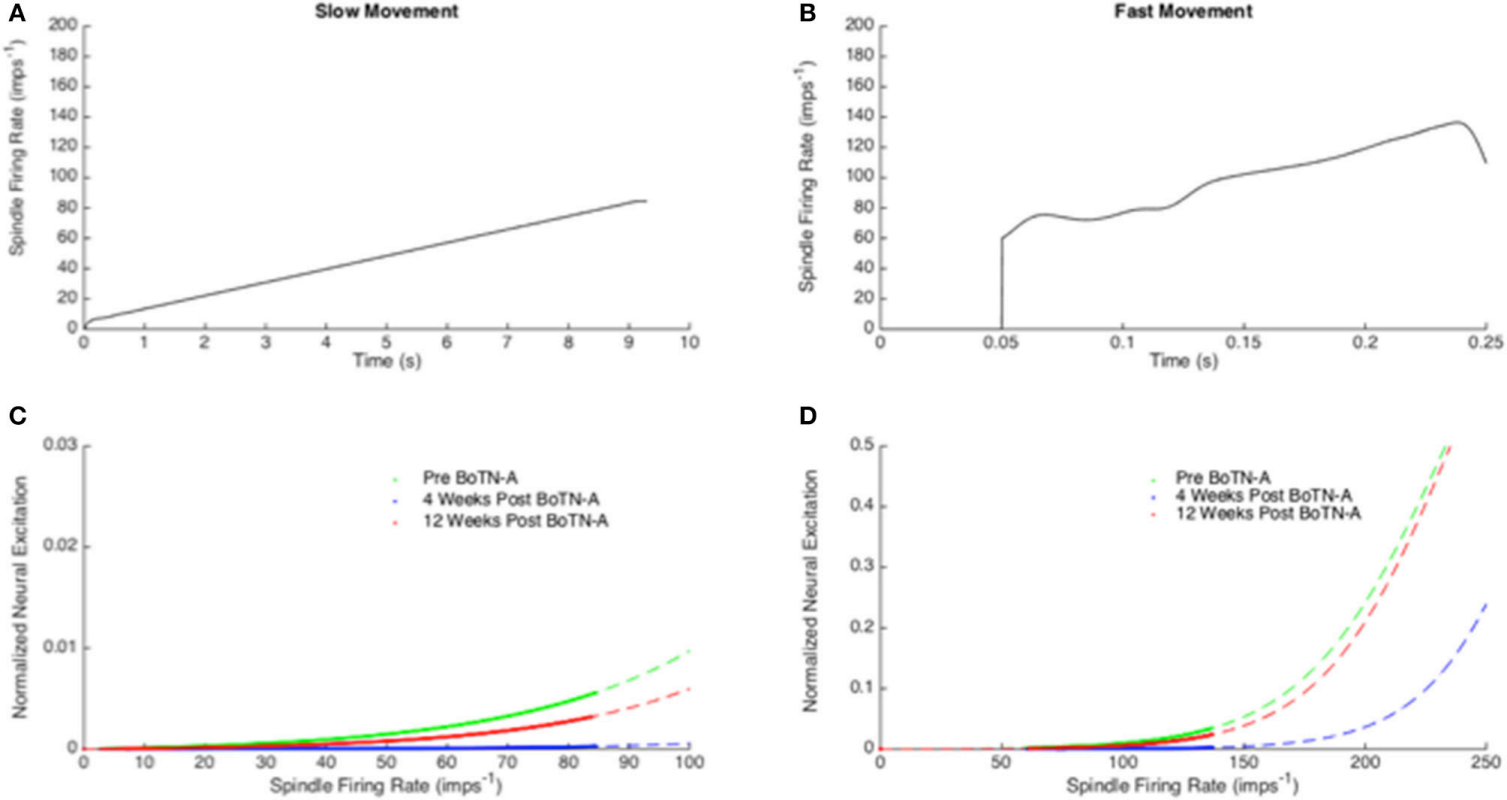
**(A,B)** Model based estimates of muscle spindle firing rate induced by wrist extension in the slow **(A)** and fast **(B)** movement. **(C,D)** Mean motoneuron pool profiles were estimated using optimization at three test occasions (before BoTN-A treatment, 4 and 12 weeks after BoNT-A treatment). The dash lines in green, red and blue represent the motoneuron pool profile in a broader range of the spindle firing rate (0 imps^−1^ to 100 imps^−1^ for the slow movement; 0 imps^−1^ to 250 imps^−1^ for the fast movement) and the solid lines correspond to the range of the neuron excitation estimated in the current study.

For two motoneuron pool parameters (μ and σ), a significant increase in μ between baseline and 4 weeks post BoTN-A (*p* = *0.01*) was observed, and a reduction of μ at 12 weeks after treatment was found, but the changes were not significant (Table 2). No significant changes were found in σ between test occasions. The participants had a significantly higher motoneuron pool threshold μ_0_ at 4 weeks post BoTN-A, but no differences were found in the motoneuron pool gain *G*_0_. Significant and high correlations between μ_0_ and *G*_0_ were determined in three test occasions (pre BoTN-A: *p*<*0.01, r*_*s*_ = 0.73, 4 weeks post BoTN-A: *p*<*0.01, r*_*s*_ = 0.75, 12 weeks post BoTN-A: *p*<*0.01, r*_*s*_ = 0.81, Figure 3). The averaged motoneuron pool profiles extracted before and after treatment are illustrated in Figure 4.

### Correlation of individual components from model estimation and the NF-method

The EC, VC, and NC were identified at the third peak of the resistant torque in the fast movement, as the same as the NF-Method. To be consistent in the unit, individual torque component from model estimation was converted to the force component with a constant moment arm (the measured distance from the force sensor to the joint axis of NeuroFlexor). There was a significant reduction in NC between baseline and 4 weeks post BoNT-A (*p* = 0.01) and a significant increase between 4 weeks and 12 weeks post BoNT-A (*p* = 0.03). However, no significant difference in NC was found between baseline and 12 weeks after the treatment. There were no significant changes in EC and VC between test occasions. The individual components calculated from the current model estimation correlated well with those based on the NF-Method in each test occasion. A significant and high correlation was determined in NC and EC, as well as moderate correlation in VC (Table 3).

**Table 3:** The correlation between components identified by the NF-Method and optimization method was determined using Spearman's rank order test at three assessment occasions.

		**Correlation**
		**Coefficient (*r_*s*_*)**
Pre BoNT-A	NC	0.82
	EC	0.84
	VC	0.74
4 weeks post BoNT-A	NC	0.85
	EC	0.85
	VC	0.75
12 weeks post BoNT-A	NC	0.82
	EC	0.82
	VC	0.75

## Discussion

In this study, a previously developed neuromusculoskeletal model was applied to simulate the passive wrist extension test of spasticity before and 4 weeks and 12 weeks post BoNT-A injections in the chronic stroke patients. By modeling musculotendon, muscle spindle, and motoneuron pool parameter explicitly, the neural and non-neural related properties at the wrist joint were estimated using optimization techniques at each test occasion. Compared to the baseline, stroke survivors exhibited decreased neural component at 4 weeks post BoNT-A injection, which returned to the baseline levels after 12 weeks. The decreased neural component was predicted by the model to stem from the increased motoneuron pool threshold. In addition, the motoneuron pool threshold and gain were found to be highly correlated in all test occasions. To the best of our knowledge, this is the first study that has attempted to investigate the underlying neural and non-neural related changes in wrist muscles up to 12 weeks after BoNT-A injection by combining motorized measurements and neuromusculoskeletal modeling.

### Individual components and comparison to the NF-method

In order to examine whether there were systematic differences in component estimation using the NF-Method and the neuromusculoskeletal model, EC, VC, and NC were calculated at the same instance as the NF-Method (at the maximal resistance torque in the fast movement, PIII). At baseline and 12 weeks after injection, NC was the largest contributor to the total resistant torque followed by EC. At 4 weeks post BoNT-A, EC instead of NC became the largest because of the significant decrease in NC. This finding was in line with the action of botulinum toxin, where the effect was expected to be maximal at 4 weeks and to diminish after 12 weeks post the injection (Gäverth et al., 2014). No significant changes in EC and VC were found over the time as expected, since BoNT-A was not assumed to influence the mechanical properties of the muscles. Moderate to high correlations with the NF-Method were found in all three components over time (Table 3). Similar correlations were reported from our previous study, though only baseline data was analyzed (Wang et al., 2017).

### Passive and stretch reflex related parameters

#### Passive parameters

The passive wrist stiffness and viscosity at baseline in the current study were comparable to our previous report (Wang et al., 2017). No comparison data were found for stroke survivors after BoTN-A treatment in the literature. Compared to the baseline, although the linear stiffness (*K*_*p*_) and viscosity coefficients (*B*_*p*_) were similar, a tendency of the increased nonlinear exponential (*k*_1_) term was observed over time and significance was determined at 12 weeks (Table 2). During the slow movement, the passive tissue demonstrated a quasi-linear resistant torque in response to the increased wrist angle. A higher non-linear coefficient (the exponential term) results in an earlier exponential increase in the resistant torque. It might indicate a reduced ROM of the wrist joint over the time, because larger resistance torque develops in the end of the range of motion, close to the participant' s maximal extension limits. Compared to the baseline, we found the passive ROM of the wrist was significantly decreased at 12 weeks, which verified our hypothesis. Shortening of the muscle and/or connective tissues often induced by longstanding spasticity is regarded as the major contributor to the passive movement limitation (de Bruin et al., 2014), which may cause an earlier start of the exponential rise of the stiffness torque. Studies on musculoskeletal stiffness after BoTN-A injection were often conducted with very different protocols, thus resulting in somewhat contradictory conclusions. Moreover, the effect of BoTN-A on passive muscle stiffness measured biomechanically in humans has rarely been reported in the literature. To our knowledge, the only study was conducted on children with cerebral palsy after an injection of BoTN-A in triceps surae (Alhusaini et al., 2011). In their study, no significant change in either passive range of movement or passive stiffness of the ankle muscles was reported 6 weeks post the injection. However, in animal models, the passive elasticity module of muscle fascicles increases 1 month post injection (Thacker et al., 2012). A recent study on rats also found that the passive stiffness of the injected limb was increased at 2, 4, and 8 weeks (Pingel et al., 2016).

#### Stretch reflex related parameters

In the current study, the modeling of the reflex loop was simplified and consisted of muscle spindle and motoneuron pool models. The static and dynamic gain (*G*_*l*_, *G*_*v*_) of the hybrid V^0.6^ model reflected the level of γ-motoneuron activation. Recent research has shown that the firing rate of muscle spindles in the stroke survivors was similar to those of the controls (Dietz, 2003). Therefore, we assumed that the hyperactive stretch reflex was mainly due to the abnormal input-output relationship of the motoneuron pool. The changes of NC were further investigated by identifying the motoneuron pool profile for each stroke survivor before and after treatment (Figure 4). By definition, all of the motor units that innervate a certain muscle, i.e., the lumped wrist flexors, are considered as a motoneuron pool. Each motor unit consists of a single motor neuron and the muscle fibers it innervates. Therefore, the overall motoneuron pool behavior reflects qualitatively the suppress effect of the BoTN-A treatment at the neuromuscular junction where synapse with the motor end plates of the muscle fibers. Significant differences were observed between the baseline and 4 weeks post BoNT-A injection (Table 2). According to our modeling and analysis, reduced hyperactive reflex was mostly due to higher μ. In order to achieve a more meaningful interpretation of each participant's motoneuron pool profile, the threshold μ_0_ (the minimal spindle firing rate) and gain *G*_0_ (the mid-range slope) were defined. Physiologically, *G*_0_ can be interpreted as an intrinsic motoneuron property, e.g., passive membrane properties and the voltage sensitive membrane conductance (Koo and Mak, 2006), and μ_0_ as a net excitatory and inhibitory inputs to the motoneuron pool. Compared to the baseline, μ_0_ had a significant increase 4 weeks after the injection and returned back to the baseline level at 12 weeks, while no significant changes in *G*_0_ were noticed over time. Similar findings using an experimental approach were reported by Stampacchia et al. (2004). In their study, they quantified the stretch reflex threshold as the sudden increase in the recorded EMG activity at the lowest wrist extension velocity. They found that the stretch reflex threshold increased after the botulinum toxin treatment. They explained that the observed increased stretch reflex threshold was mainly due to the intrafusal action of the drug, e.g., the reduced afferent discharge and stretch sensitivity in the muscle spindles. Theoretically, neural excitation can be enhanced by reducing μ_0_ without changing the shape of the profile (*G*_0_), i.e., shifting the profile curve to the left. However, we found high correlations between μ_0_ and *G*_0_ in all test occasions (Figure 3), in line with our previous study (Wang et al., 2017). This finding agreed with our previous assumption that the abnormal motoneuron pool excitability was possibly modulated through a combined effect from the overall synapse input and the intrinsic properties of motoneurons. However, under the intervention of BoTN-A, the dominant effect was from the former factor, i.e., the overall synapse input represented as μ_0_ in our modeling approach. A significant increase in μ_0_ at 4 weeks implied that less motor neuron could be excited after the BoTN-A injection. This is also in line with the pharmacological mechanisms of BoTN-A, which inhibits the neural transmission at the neuromuscular junction.

### Clinical implications

In combination with the NeuroFlexor measurement, a computational model accounting for musculotendon, muscle spindle, and motoneruon pool effects can help identify potential neural and non-neural related parameter changes along the stretch reflex pathway in the spastic patients after treatment of BoTN-A. These parameters are physiologically meaningful, but not directly measurable. Moreover, this approach can also be applied in other non-pharmacological interventions, e.g., physical interventions and orthotics. Monitoring neural and non-neural related parameters prior to and post treatment can provide insights into different intervention strategies. In addition, our findings can be used to improve the measurement protocol of the NF-Method. The current measurement protocol operates in a fixed ROM, which excluded the severely affected patients with limited ROM. Consequently, the estimation of EC was not sufficiently accurate to describe passive stiffness in each participant's full ROM. An individualized ROM would be more informative and appropriate for this heterogeneous population.

## Limitations

Several simplifications were made in the model. First, the optimization was only performed in part of the fast movement. There is a short “mechanical chaotic state” after the first acceleration peak in the fast movement, which occurs around 0.06–0.07 s. During that period, the force sensor is unloading due to a small gap between the handplate and the force sensor. Thereby, no valid force data was collected. The improvement of the mechanical design of the NeuroFlexor is valuable in the future. Second, the goal of the current study was to use a general but valid forward neuromusculoskeletal model and optimization framework that allows for reliable estimation of passive muscle-tendon and stretch reflex-related parameters before and after the BoTN-A treatment in each person. However, the authors are aware that neurophysiological measurement, e.g., EMG activity, can provide more information of spasticity when combined with a resistance measurement. In addition, NeuroFlexor is not able to separate finger flexors and wrist flexors and our analysis cannot identify parameters on an individual muscle basis. Finally, the sample size of the patients is small and there was several drop-outs at 12 weeks. Future studies with larger cohorts will improve the generalization of our findings to the entire stroke population.

## Conclusions

Neural and non-neural related properties of wrist flexors were estimated in persons with chronic stroke before and after BoTN-A injection using a forward neuromusculoskeletal model and optimization. The model describes the overall resistant behavior of the wrist joint during a motorized extension test of spasticity. Compared to the baseline, the neural contribution to the decreased resistant torque at 4 weeks was predominated by the increased stretch reflex threshold in the model. Moreover, an increased non-linear stiffness coefficient indicated a reduced wrist ROM over time, even though the treatment effect of reducing spasticity was observed. We concluded that in combination with the NeuroFlexor measurement, the proposed neuromusculoskeletal model and optimization scheme served as suitable tools for investigating potential parameter changes along the stretch reflex pathway in persons with chronic stroke. These neural related parameters are physiologically meaningful but not directly measurable. By monitoring their values before and after interventions, (e.g., BoNT-A treatment and other therapies), valuable insights can be provided for effective treatment response.

## Author contributions

RW conceived the model, developed it, analyzed the data. PH developed optimization framework. JG collected the data. All authors have been involved in manuscript preparation.

### Conflict of interest statement

Author JG owns part of the commercial rights of the measurement instrument described in this study as shareholders of the manufacturing company Aggero MedTech AB. The remaining authors declare that the research was conducted in the absence of any commercial or financial relationships that could be construed as a potential conflict of interest.
